# The Daily Mile as a public health intervention: a rapid ethnographic assessment of uptake and implementation in South London, UK

**DOI:** 10.1186/s12889-019-7511-9

**Published:** 2019-08-27

**Authors:** Benjamin Hanckel, Danny Ruta, Gwenda Scott, Janet L. Peacock, Judith Green

**Affiliations:** 1 School of Population Health & Environmental Sciences, King’s College London, London, UK; 2 Public Health, London Borough of Lewisham, London, UK

**Keywords:** Children, Physical activity, Schools, Implementation, Rapid assessment, Qualitative research, The daily mile, Intervention fidelity, Adaptation, Context

## Abstract

**Background:**

Existing evidence identifies health benefits for children of additional daily physical activity (PA) on a range of cardiovascular and metabolic outcomes. The Daily Mile (TDM) is a popular scheme designed to increase children’s PA within the school day. Emerging evidence indicates that participation in TDM can increase children’s PA, reduce sedentarism and reduce skinfold measures. However, little is known about the potential effects of TDM as a public health intervention, and the benefits and disbenefits that might flow from wider implementation in ‘real world’ settings.

**Methods:**

We aimed to identify how TDM is being implemented in a naturalistic setting, and what implications this has for its potential impact on population health. We undertook a rapid ethnographic assessment of uptake and implementation in Lewisham, south London. Data included interviews (*n* = 22) and focus groups (*n* = 11) with stakeholders; observations of implementation in 12 classes; and analysis of routine data sources to identify school level factors associated with uptake.

**Results:**

Of the 69 primary schools in one borough, 33 (48%) had adopted TDM by September 2018. There were no significant differences between adopters and non-adopters in mean school population size (means 377 vs 397, *P* = 0.70), mean percentage of children eligible for free school meals (16.2 vs 14.3%, *P* = 0.39), or mean percentage of children from Black and Minority Ethnic populations (76.3 vs 78.2%, *P* = 0.41). Addressing obesity was a key incentive for adoption, although a range of health and educational benefits were also hypothesised to accrue from participation. Mapping TDM to the TIDierR-PHP checklist to describe the intervention in practice identified that considerable adaption happened at the level of borough, school, class and pupil. Population health effects are likely to be influenced by the interaction of intervention and context at each of these levels.

**Conclusions:**

Examining TDM in ‘real world’ settings surfaces adaptions and variations in implementation. This has implications for the likely effects of TDM, and points more broadly to an urgent need for more appropriate methods for evaluating public health impact and implementation in complex contexts.

**Electronic supplementary material:**

The online version of this article (10.1186/s12889-019-7511-9) contains supplementary material, which is available to authorized users.

## Background

Physical exercise in childhood is important for children’s current health and wellbeing, and for their future health outcomes [1]. Lower rates of physical activity have been associated with cardiovascular and cardiometabolic risk factors [2, 3], and poorer mental health outcomes [1, 4]. Lack of moderate to vigorous physical activity (MVPA) has also been linked to overweight and obesity in children [5], although there are challenges in identifying the direction of causation [6]. In the UK, the National Child Measurement Programme (NCMP) has identified increasing numbers of overweight and obese children in England, with enduring inequalities by area deprivation and region in all countries of the UK [7, 8]. There are challenges in reliably recording rates of MVPA in children [9], but indicators suggest that few children in the UK meet recommended targets for MVPA [10, 11]. There are then, good grounds for developing and promoting interventions which can increase the amount of physical activity, particularly MVPA, that children undertake.

The Daily Mile (TDM) is one promising intervention for increasing physical activity in children. Originating in Stirling, Scotland, in 2012, the TDM initiative requires school teachers to take schoolchildren out of their classroom to run for 15 min per day, which equates to a distance of approximately one mile. TDM is a scheme promoted by The Daily Mile Foundation, a non-profit organisation funded by INEOS, a multinational petrochemicals company. According to the Foundation website, the aims of TDM are to improve the ‘physical, mental, emotional, and social health and wellbeing’ of children [12]. Teachers can implement it at any time of the day, and in varied weather conditions, without any need for special equipment. It is therefore designed to be a simple, free (in principle) and sustainable intervention which is inclusive for all children, including ‘children with mobility difficulties [who] should be fully supported to take part as well’ [12]. The scheme has been acceptable to schools, parents and children, and has now been taken up increasingly across the UK, and beyond, with considerable policy support [13]. As of February 2019 there are over 7000 schools and nurseries taking part, with over 4000 of those schools in the UK [12]. TDM is in principle easy for schools to implement requiring limited time out of class, no special clothing, and no staff training. Indicative results from a pilot study of TDM in Stirling found that children in an intervention school had increased the number of minutes per day of MVPA, increased physical fitness and decreased skinfold measures compared to children in a non-intervention control school [14]. One RCT of TDM is already underway in Birmingham [15], which will provide invaluable evidence on impact - including BMI, quality of life, wellbeing, and academic attainment - albeit in the context of a trial in one region.

There is, then, good evidence that increases in MVPA will improve children’s current and future health [1], and that taking part in TDM can contribute to such an increase in principle [14]. However, there is no robust evidence to date of whether rolling out TDM in schools more generally is likely to have a positive impact on the public health and what the un/intended impacts of the intervention outside of trial studies/RCTs might be. This study contributes to this evidence base by examining how TDM is implemented in a ‘real world’ setting. Examining public health impact in ‘real world’ settings is crucial as pilot trials or RCTs are often unrepresentative of everyday practice: there may be increased input to achieve fidelity; participants in trials may be more committed to the intervention, and context plays an important role in shaping the intervention [16]. Impact is here thus understood through ‘real world’ settings of adoption (e.g. schools), as well as the implementation consequences, both the intended and unintended ones, and the ongoing efforts to maintain or sustain the intervention, within the contexts in which they are situated [17]. Often RCTs and pilot trial data cannot account for the ‘moderating factors’ of interventions in real-world settings, which can create difficulties for the transferability of RCTs to practice settings [18].

There has been some scepticism from the public health community on both the likely effects and sustainability of TDM, citing concerns about the minimal impact of an additional 15 min of activity, concerns about it displacing more effective forms of exercise in the school day (such as active play), and the risks of putting children off future participation in sports and exercise if the activity does not meet their needs for meaningful activity [19]. Despite its perceived simplicity and the fact that it is promoted as ‘free’, the scheme has a number of components (time, organisation, staff input, a safe accessible space to run), which must be assembled for the intervention to be implemented. As in other school-based interventions [20], there is likely to be considerable variation in implementation across school settings, and it may be adapted in various ways by individual class teachers and participating pupils. Schools not adopting TDM may be taking part in other, similar schemes. Individual children taking part may or may not be also participating in other forms of physical activity, or broader healthy schools initiatives. These factors present significant evaluation challenges in identifying the effects of TDM as a single intervention, and in identifying what the essential components of an effective intervention might be.

The focus of this study is to understand how TDM is being implemented in practice, and to provide a firmer foundation for future evaluations of the public health impact of this and similar interventions. Our aims thus were to: identify factors that impact on whether TDM is adopted in particular settings (e.g. schools and classes); to describe how TDM is being implemented; to identify intended and unintended consequences; and to describe factors that affect whether implementation is maintained. A secondary aim of the study was to identify potential design considerations for future evaluation.

## Methods

Below, we outline the study design, and provide an overview of study participants and our analysis. We use the COREQ checklist [21] to guide reporting.

### Study design

To understand how TDM operates as a public health intervention in a naturalistic setting, we undertook a study in 2018 (with fieldwork conducted between May and December) of what happened when the scheme was promoted for primary schools (for children aged 5 to 11) across one local authority area, the south London borough (LB) of Lewisham. To understand adoption and implementation of TDM in Lewisham, we undertook a rapid ethnographic assessment [22]. This is a pragmatic, focused and mixed-method approach, using analysis of data from mixed-methods (observations, interviews and focus groups, as well as secondary data analysis) to generate evidence for evaluation [22, 23].

### Setting and population

Lewisham is a diverse borough of south London, UK, with 29.6% of children living in income deprived households [24]. The borough includes 69 primary schools in the state sector educating pupils aged between 5 and 11 years. These schools range from small stand-alone primary education providers to larger federations of schools which include primary years. The Health & Wellbeing Strategy in Lewisham has ‘achieving a healthy weight’ as a priority area for action, and its Public Health Report of 2016 [25] set out a whole system approach to obesity which included supporting three key initiatives: Sugar Smart (a campaign to reduce sugar in diets); greater use of parks within the borough; and uptake of TDM across its primary schools. This intervention (TDM) was therefore being supported by the public health directorate, who were encouraging schools to sign up.

### Sampling strategy and procedure

To examine experiences in south London we used a purposive sampling approach to identify and select stakeholders and cases (i.e. schools) from the locality who had varied experiences with TDM adoption and implementation. This included Lewisham public health practitioners (*n* = 3), who were interviewed about their experience implementing the project in the borough. Lewisham Public Health also provided data about school characteristics, TDM adoption, percent of pupils from Black and Minority Ethnic (BME) communities and percent of pupils eligible for free school meals. Recruitment was undertaken through invitations sent through a monthly public health newsletter to schools, direct emails to head teachers, as well as recruitment at a local event focused on physical education and sports in schools. Of those schools who indicated an interest we initially selected 6 schools, with varying levels of success in rolling out the intervention. However, one school chose not to participate, with 5 schools in total participating in this study. The schools included a range of school types (e.g. faith schools, community schools) with varied levels of TDM implementation (see Table 1).

**Table 1:** Schools in the Qualitative Component of the Rapid Ethnographic Assessment

	School Implementation	Free School Meals (%)	BME (%)	Faith-school
School01	3 years or less, Whole School	17.1	89.8	Yes
School02	2 years or less, Select Classes	9.5	63.1	No
School03	2 years or less, Select Classes	16.4	87.1	Yes
School04	2 years or less, Select Classes	25.1	77.5	No
School05	Ad hoc, Select Classes	22.9	73.5	No

### Qualitative data generation

Qualitative data were collected at each school. This included in-depth interviews (*n* = 22); focus groups (*n* = 11) with 41 participants; and participant observation of 49 Daily Miles across 12 classes in the five schools. Interviews and focus groups were recorded and lasted for approximately 30–45 min in length. All participants self-selected to take part and provided their own and, where applicable, parent/guardian consent. Participants from the 5 schools did not know the researcher prior to the commencement of the study.

Of the 63 participants who participated, in-depth interviews were undertaken with public health practitioners (*n* = 3), headteachers (*n* = 2), assistant head teachers (*n* = 2), school teachers (*n* = 5), and pupils (*n* = 10). Focus groups took place in school settings with teaching staff (assistant head teacher (*n* = 1), school teachers (*n* = 2)), parents and carers (*n* = 3), and pupils (*n* = 35). Focus groups were made up of participants of the same gender (*n* = 5), as well as mixed gender (*n* = 6).

Of the qualitative sample, in total 56% of participants were female and 44% male. The majority of teaching staff (*n* = 11) and public health practitioners (*n* = 2) identified as female. All parents and carers (*n* = 3) identified as female. There were slightly more male children (*n* = 25), than female children (*n* = 20). Children ranged in ages from 7 to 8 (16), 9–10 (17), and 10–11 (*n* = 12). All teaching staff interviewed were white, whilst 20 children, and 1 parent were BME.

Interview/focus group schedules were semi-structured and covered similar themes with all participants. This included questions related to their involvement in TDM, what worked well, any challenges and costs associated with the implementation of the programme, and, (for public health professionals and school staff) what medium-long term plans were in place to sustain the intervention (if any) (See Additional file 1 for copies of the interview/focus group schedules).

Observational data included 61 h of observations across all schools. Field notes recorded transitions from classroom to TDM, the implementation of TDM, and transition back into the classroom afterwards by the lead author (BH). Field notes were guided by both the implementation intended outcomes, and topics that emerged from in-depth interviews and focus groups. Field notes were recorded before, during and after the implementation of each TDM across a two-three week period in each of the 12 classes to document what happened in practice.

### Quantitative data sources

Routine data for schools in Lewisham were collated from existing records, which included secondary data held by Lewisham Council and data they collected as part of the roll out of TDM in the Borough. Existing data on school characteristics of all schools included size of the school (number of pupils); per cent of pupils with free school meal entitlement; and per cent of pupils who identified as from BME populations. The Department of Public Health also recorded date of adoption of TDM.

### Analysis

Interview and focus group data were transcribed verbatim. All qualitative data, transcribed interview and focus group data, as well as fieldnotes, were analysed by researchers using thematic content analysis [26], using NVivo software.

For analysis of routine data on school uptake we calculated summary statistics in the two school groups (‘daily mile implemented’ and ‘no daily mile in school’). All data were continuous and these were therefore summarised with group means and standard deviations. The distributions of each variable were clearly non-normal (number of pupils was bimodal, percent pupils with free school meals was positively skewed, and percent pupils BME was negatively skewed). To compare the distributions of these characteristics in adopting and non-adopting schools, we used two-sided Mann-Witney U tests since t tests would not be valid with bimodal and skewed data. We drew density plots to depict the overlap of the distributions of characteristics by school group. The statistical data analysis was conducted using STATA 14.

To describe the variation in implementation, we used the template for intervention description and replication for public health and policy interventions (TIDieR-PHP) [27]. This was developed from the existing TIDieR checklist [28] to improve the reporting of key features of interventions, such that they can be replicated. Items 9.1 and 9.2 of this checklist refer to fidelity: how well this was assessed and maximised (item 9.1) and how well it was actually achieved in delivery (item 9.2). These are perhaps most relevant for trial designs, rather than observational designs in contexts such as this, where the roll out of TDM aimed to encourage as many schools to sign up as possible, rather than to ensure that they were adhering to the core principles promoted by TDM Foundation. Issues of fidelity are also problematic in observational designs such as rapid assessments, where the aim is observing what happens in practice in non-trial settings, rather than necessarily intervening to maximise fidelity. We therefore used item 9.2 from the TIDieR checklist as a way of summarising how TDM was implemented in practice in schools across Lewisham.

## Results

### What factors were associated with uptake of the intervention?

Of the 69 schools with primary years provision in the borough, 33 (48%) had adopted TDM by September 2018. Five of these had been taking part since 2016, 17 had started in 2017, and six during 2018. The summary statistics show that the 33 schools that adopted TDM had a lower mean number of pupils with a narrower range of sizes compared to those who did not adopt TDM (*n* = 36) but the differences were not statistically significant (*p* = 0.70). TDM schools had a slightly greater mean percentage of pupils having free school meals than the non-TDM schools but this was not significant (*p* = 0.39). The mean percentage of pupils of BME groups were similar in the two school groups (*p* = 0.41). No differences in characteristics were statistically significant (Table 2).

**Table 2:** Summary statistics of participating and non-participating schools

	Daily Mile implemented in School *N* = 33	No Daily Mile in School *N* = 36	*P* value (2-sided) (Mann-Whitney U test)
Number of pupils			
Mean (Std Dev)	377 (128)	397 (147)	0.70
Range	204 to 645	192 to 665	
Percent with Free School Meals			
Mean (Std Dev)	16.2% (8.5%)	14.3% (6.7)	0.39
Range	4.1 to 41.6%	4.2 to 31.2%	
Percent pupils BME			
Mean (Std Dev)	76.3% (13.0)	78.2% (15.1)	0.41
Range	53.1 to 99.7%	42.5 to 97.6%	

**Fig. 1:**
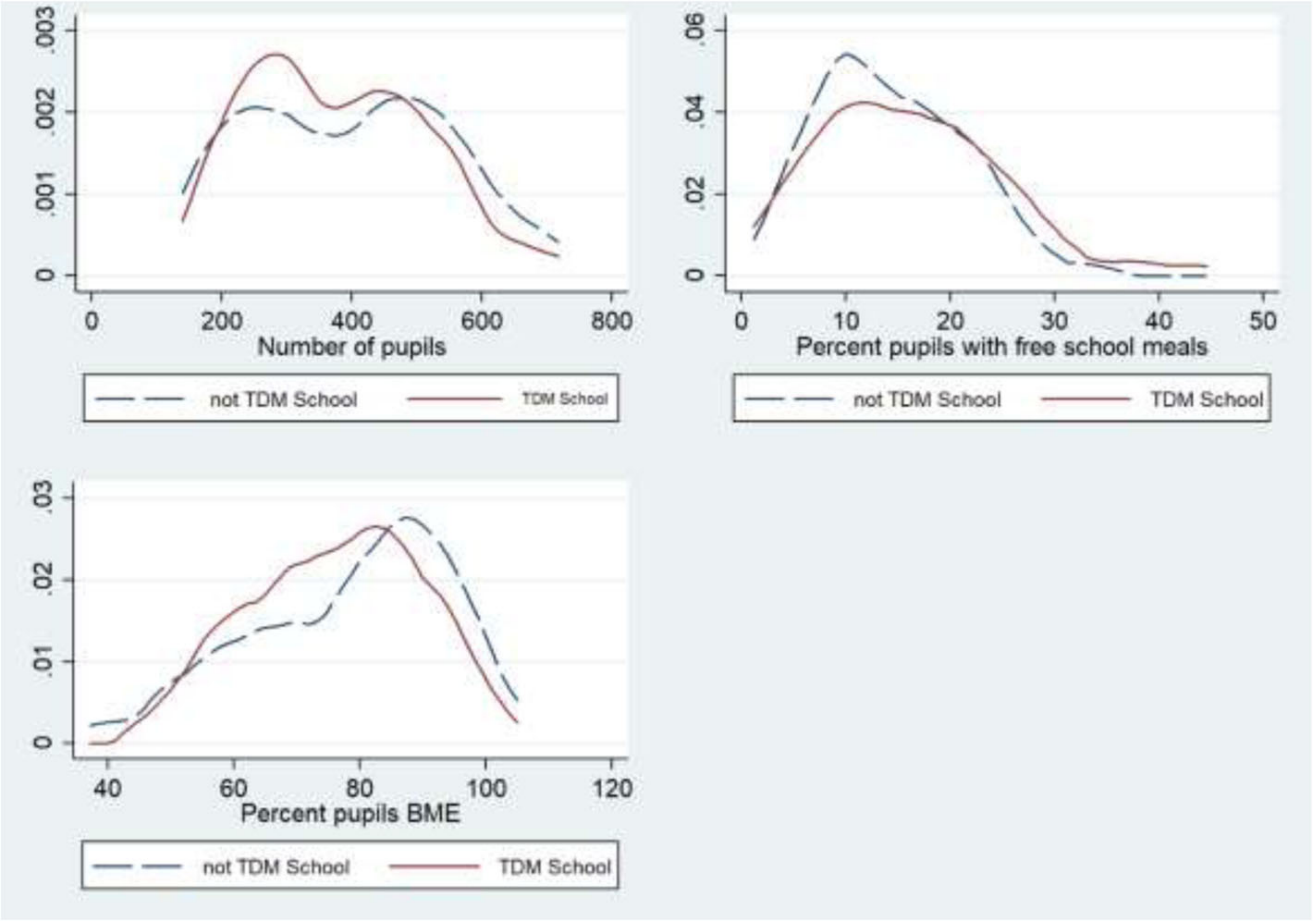
Comparisons of adopters (TDM School)/non-adopters (not TDM School) by number of pupils, percent free school meals, percent Black/Minority Ethnic (BME)

Figure 1 (see below) confirms visually the high variability among schools and shows the overlapping distributions for each of the three characteristics described above.

### The intervention in practice: how the daily mile was implemented in Lewisham

Within schools who did adopt TDM, uptake varied across classes. In some schools, all classes were taking part, in others, this included only select classes. Regularity varied by both school and often classroom-to-classroom, and the actual practice (what ‘doing TDM’ meant) varied considerably.

Table 3 (below) summarises the description of TDM as an intervention ‘as intended’ (that is, as described on the Foundation’s website and supporting materials) and ‘as implemented’ across items 1–8 of the TIDieR-PHP checklist.

**Table 3:** Summary of TDM as implemented in Lewisham, using the TIDieR-PHP checklist

TIDieR- PHP Item	As described by TDM Foundation^a^	How TDM was implemented in Lewisham (Items 9.2: Fidelity)
1. Name	The Daily Mile	The Daily Mile On occasion other terms used by schools, such as ‘The Daily Run’ or ‘The Daily Stroll and Chat’.
2. Why: logic, mechanisms or goals of intervention	To increase children’s physical, mental, emotional and social health and wellbeing	The public health team, teachers and heads focused on the potential to reduce childhood obesity. Teachers and heads also emphasised TDM as a potential ‘corrective’ to health disadvantage from deprivation.
3. What materials	Outdoor space, a ‘firm and mud free surface’ and a route that has been risk assessed No special clothing or equipment needed	Participating schools generally had this, either in playgrounds or nearby parks, but some had very restricted outdoor space, with physical barriers (such as several flights of stairs; lack of outdoor space).
	‘Transitions between class and route should be slick’	Classes must navigate with other classes undertaking TDM, or regular PE, and have to coordinate around these other activities as space is often limited.
4. What and how	TDM aims to include whole classes in a daily run (or jog) (self-paced) for 15 min per day, outdoors, within the school day	Variations in who walked, ran, jogged. Variations in delivery – some classes developed games, which included a mixture of walking, running and sitting at different intervals. Reductions in TDM time if class curriculum runs overtime. Not all classes were running a mile (some walked, or engaged in class activities that required some physical movement), and it often did not conform to only 15 min per day.
	‘Social, non-competitive, fun’	Many children (and some teachers) introduced elements of competition.
	‘They can chat to their friends as they run along enjoying the experience together.’	‘Chatting’ often perceived as a negative by staff and associated with walking: it is often not seen as an activity that is congruent with running and completing the mile and/or increasing fitness/stamina.
5. Who provided the intervention	Head teachers sign up school TDM requires no particular training, but TDM Foundation website (https://thedailymile.co.uk/) provides information and resources such as promotional material for those signing up	Lewisham public health department provided considerable input, including: organising initial meetings and a schools conference with the TDM founder; 2 surveys to generate interest from schools; regular promotional mailings to schools; ongoing phone calls and visits; providing case study materials; including information with the NCMP letter to schools and information in a school governor pack.
	Teachers go out with their class	Teachers did generally go out with their class; some ran, some stood and watched. In general, other class staff (e.g. teaching assistants) did not participate.
6. Where	Primary schools (ages 5–11) It can also be done in early years settings. Started in Scotland, now in operation across Europe and beyond.	48% of primary schools in Lewisham had adopted the scheme, and at least one Year 7 class in a secondary school.
	Outside in fresh air	Low air quality levels in some areas of Lewisham reduce access to fresh air.
7. When and how often	Everyday (in practice ‘at least 3 times a week’), whatever weather, 15 min	Not every day in most schools/classes. Seen as interchangeable with other physical health interventions adopted within the school; only undertaken on non-PE days in some schools; depends on busy periods. May take longer than 15 min; sometimes less time. Weather was an inhibitor at times, and TDM not undertaken when considered ‘unsafe’ or too wet.
	During curricular time	At times TDM is not implemented as the curriculum for the day is considered ‘too full’.
8. Planned and unplanned variation	Inclusive: all children	All children were included, but there were gender differences in how it was adopted by participating children. Girls observed and reported to be more likely to walk and ‘chat’.
	‘keep it simple’	Many teachers initiated games to keep it interesting.

^a^Quoted material from the TDM web site ‘Core principles’ and other pages https://thedailymile.co.uk/steps-to-success/ as of October 10th 2018

In practice, TDM was adapted at several levels. The local authority promoted uptake of TDM through organising meetings for school leaders, including visits from the founder of TDM, promotional mailings, and supporting schools by identifying ‘pioneers’ who could share their experiences with other schools. Schools themselves adapted the scheme; classes within schools differed in what they did, and there was variation at pupil level.

#### The intervention name

Locally, within the borough, the scheme was being promoted as ‘The Daily Mile’, as described in the TDM Foundation website. Some schools had adopted different names, such as ‘The Daily Run’ (to emphasise that children did not have to cover a mile, as some teachers were concerned that this would take longer than the 15 min scheduled and disrupt class time).

#### How the logic and mechanisms were understood

Although TDM Foundation website highlights the general wellbeing benefits, the LB of Lewisham promoted TDM primarily as a part of their wider strategy around obesity. The opening line of their policy document supporting the scheme was “Lewisham, in common with most of the country, and in particular London, is undergoing an obesity epidemic”. Concern about local levels of obesity were also reported as a key incentive for adopting TDM for heads and teachers in local schools, who linked their enthusiasm to efforts to reduce risks of obesity:

the government wants us to address obesity in primary age children, and as a school we were seeing an increase in children … with very worrying weight problems, and they weren’t really being addressed, although the school does have healthy lunch policies, we’re a healthy school, we only allow fruit, we don’t have tuck shops, but it wasn’t enough. So we needed something else. (Participant 13, Teacher)

Children also saw these links between weight loss, participation and fitness (“if you are very tubby, or fat, or whatever, then I recommend the Daily Mile to you to get fit” Participant 36, Student). For males this was often linked to gaining strength (“if you’re not fit it can affect your body sometimes … It can make you weaker” Participant 55, Student). For other children it was linked to bullying avoidance (“so if you’re overweight you would burn down any calories to make you like thinner and thinner so you not always get called stuff” Participant 27, Student).

A wider range of benefits from engaging in the scheme were also identified by participating teachers and heads. The benefits were most pronounced, they argued, for children who might not get much other physical activity in their day (“I think the less sporty ones are benefitting more … even though they can do it consistently, there’s more of a noticed progress with the less engaged children”, Participant 06, Teacher). Parents and carers indicated similarly:

I think they all benefit, but I’ll be honest there are some children that do have some weight issues, I’m not going to lie, as I said my son’s not the slimmest, and he’s not the fittest, but I think they all benefit from it, but some more than others (Participant 09, Parent)

A key benefit was improved physical fitness for children, and teachers often adopted ways of monitoring or demonstrating this as an outcome of children’s participation, through (for instance) recording the number of miles children had run (“we have a big number on the board of how many miles we’ve done each day and we keep data over the week”, Participant 05, Assistant Head Teacher) or utilising tools such as “a heartrate monitor … to show how … increased activity and intense activity raises their own heart rates” (Participant 13, Teacher), or similarly getting students to monitor their own heartrate before and after engaging in TDM. As one teacher explained:

[I say to the class] ‘Feel your pulse, right, let’s make our hearts beat faster, that means going … our bodies are working harder, and that’s going to be making us healthier’ (Participant 62, Teacher)

These activities were seen to both encourage students to participate actively in the TDM initiative, but also contribute to their physical literacy; a further outcome anticipated from taking part. The link anticipated between TDM participation and health, or health literacy, was confirmed in interviews and focus groups with children (“because you feel your heart is racing and it’s burning all the fat that you’re eating … so your heart races, you feel healthier”, Participant 26, Student).

Teachers also identified important non-health benefits. When the intervention took place after lunch it had the reported effect of addressing tensions between students to “run off any problems”(Participant 06, Teacher), it was positive for peer relationships between children, within their class, as well as peer relationships across year-groups, which we observed as classes across the school would undertake TDM together. It was also cited by teachers to be positive for teacher-student relationships:

it’s also quite nice to get to know some of the children … they just talk to you about stuff, and you would never really have that opportunity in any other time (Participant 05 Assistant Head Teacher)

In addition teachers indicated it contributed to, at times, better concentration in class, and made contributions to other parts of the curriculum, which they believed enhanced learning outcomes. They reported using TDM as an opportunity to discuss “geography … and of course numbers, [and] maths … I talk to them about, you know, well in a week what sort of average are we doing” (Participant 04, Headteacher), or opportunities to link to wider learning:

we’ve learnt shapes of leaves, we’ve done collections for artwork via the Daily Mile, so we might bring bags with us one day and collect things for art, or DT [Design Technology], or make things in the park, so we’ll go the park, we’ll use the resources, photograph them, and then they go on. Yeah, if you’re going to go out and do a mile, every day, you may as well bring as many elements of the children’s learning into it as possible (Participant 13, Teacher)

Few interviewees explicitly identified negative potential impacts, other than a shared concern about fitting TDM around “a timetable that’s too full” (Participant 14, Assistant Head Teacher), which led to some teachers actively choosing not to participate, or prioritising classwork over taking the class outside for a full 15 min period. In often congested areas of inner London, some teachers mentioned pollution as a general risk factor for children’s health locally, but high levels of pollution were not mentioned specifically as putting children’s health at risk when doing outdoor activity. Interviews identified possible stigmatising discourses around overweight and those ‘less sporty’, as indicated above.

For some teachers, particularly in schools with high numbers of pupils from lower socio-economic status households, TDM was suggested as a way of countering structural disadvantage, given that their pupils were framed as in higher ‘need’ of health promotion interventions to address poor health status.

So everyone’s getting something out of it, however, children that tend to live in say flats, that don’t have garden space, that don’t go out to the park, their weekly news, which is “I went to Saver Centre” week-after-week-after-week, they benefit a great deal (Participant 13, Teacher)

#### Materials

In principle, TDM does not require any particular equipment or special surfaces to run on. Most participating schools had playground facilities on which, for some, tracks had been painted, or they used nearby parks. There are now a number of commercial companies offering ‘Daily Mile’ track installation; one school had used a playground upgrade as an opportunity to install a special track. Getting to local parks could involve crossing roads, meaning that a 15 min activity would take around 30 min from class time in practice. Some Lewisham schools were in very congested sites; one had constrained outdoor space that meant doing TDM involved running around a small playground many times. Another had implemented a short ‘circuit’ of activities to replace running for 15 min, because of space constraints.

Schools in older buildings - particularly where children had to go down several flights of stairs to get outside, or which had limited outdoor space nearby – had greater challenges in managing the logistics without taking too much time from the school day. Whilst climbing stairs may have an added benefit for children in terms of PA, we found it might be more likely to have an impact on whether or not the intervention takes place at all:

proximity to the playground [is an issue] because if you’re on the ground floor it’s a whole lot easier and less time-consuming to nip out, do a quick Daily Mile and then come back, but if you’re on the top floor … coming all the way downstairs, you’re then doing your Daily Mile, then you’re going all the way back upstairs again, and so I think it’s a bit laborious if you’re on the top floor (Participant 11, Assistant Head Teacher)

#### What and how: making the TDM work for different classes and children

TDM is intended to be inclusive and non-competitive. Although schools and classes implementing the scheme did, in general, include all children in each class, in practice, games and competition were typically introduced to make the activity more appealing to some children. For instance, some teachers described how they would run alongside students and encourage them to run faster, “to spur them on” (Participant 05, Assistant Head Teacher), which we observed in practice at all schools. Some teachers developed competitive activities to encourage increased participation of children:

we came up with a thing where the class started on 10 points, if I pass them they lose a point, and if they pass me they gain 5 points (Participant 06, Teacher)

**Fig. 2:**
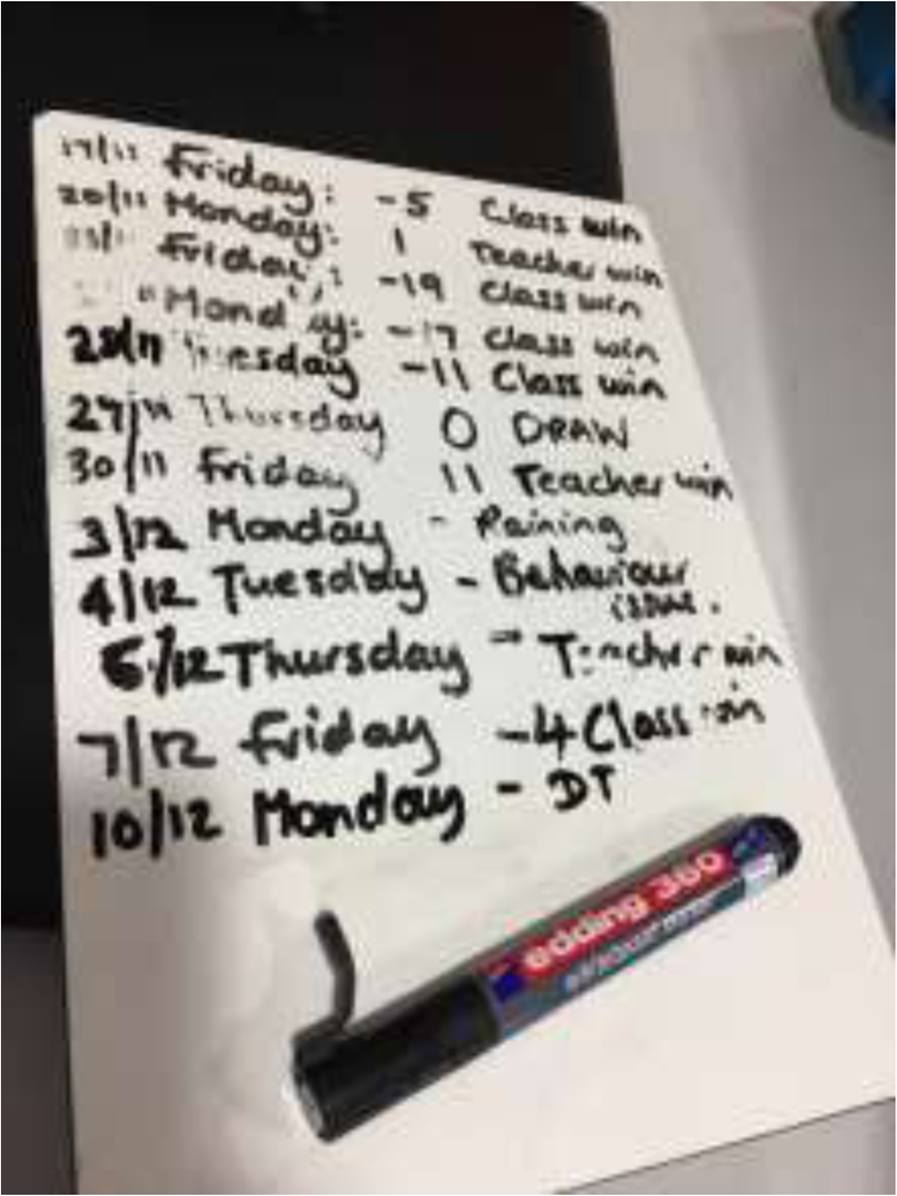
The small hand held whiteboard that the teacher takes out with the class during TDM

This was observed across several classes, and illustrated in Fig. 2 (below):

Children indicated they enjoyed these games:

The bit that we enjoy the most is when the teacher chases us around, and we, I mean we like beating her, sometimes she beats us (Participant 57, Student)

Observations and interviews identified games that had been adapted for TDM which involved competitive elements, where children compete against either their own records, or other children within the class. Teachers created PA-based games for the children to keep them engaged in the mile for 15 min. We observed children taking on different animal characters in one game to chase each other, which the teacher facilitated. These adaptations often involved planning by the teacher, and coordination, and were often used with the younger children to keep them engaged, and as a way to regulate the class during certain problematic weather conditions. As one teacher indicated during the winter:

… some days would be too cold, I mean you wouldn’t say it’s too cold for Daily Mile … it was slippery or something, so we would turn it into a penguin march but still Daily Mile (Participant 12, Teacher)

In practice, children engage in a mixture of movements during the 15 min period. In our observations we saw children moving between running, walking, skipping, and sprinting across the circuit that was established for TDM activity. This can involve stopping at times as well – some children might choose to sit down for short periods because they ‘are feeling tired’, or alternatively the teacher will actively ask them to stop between the TDM substitution activities - the games – they are facilitating. For those children who do choose to sit down we observed them moving again after a short rest, and they continue engaging in a combination or running, walking and/or sprinting, often with a group of peers.

#### Where and when

In interviews teachers often reported undertaking The Daily Mile 2–4 times a week. It was less common to implement TDM every day:

Well I think it’s that timetabling you know, Year 2 we had SATS, we had all sorts of things going on and although I know it’s beneficial, if I feel that well ‘5-a-Day’ [another PA intervention] is quite sufficient now timewise then that’s fine … we definitely do it at least, you know … three or four times a week (Participant 12, Teacher)

In part, as the interviewee describes, this is about fitting it into the curriculum, in this case making space for it in the demands/expectations of student national testing (‘SATs’), is about time demands, and has been found elsewhere to be a major barrier to implementation of health interventions in classrooms [29]. On occasion, TDM replaced other activities (such as playtime), and was often not done on days with scheduled Physical Education (PE). Often though the implementation of TDM was dependent on a teacher’s schedule, as well as other school-based activities, such as school assembly and/or excursions. Other studies have found weather to be a challenge in routinely adopting outdoor activity in schools [30]. In this setting, however, weather was only seen as an inhibitor to TDM if it was considered unsafe (“it was so icy that it was unsafe”, Participant 04, Headteacher), or if it was “pouring with rain” (Participant 12, Teacher), which is evident for instance in Fig. 2, where one day TDM did not go ahead as it was “raining”. In observations we did witness schools not participating because it was too wet, and we were also told that TDM was cancelled because a playground was flooded; in discussions with teachers, these occurrences were perceived as rare. Alternative arrangements would often be made if it was raining, such as an activity indoors, or alternatively teachers would wait until the rain stopped. In observations substituting with an alternate indoor activity was not undertaken if they were already participating in PE curriculum that day. During observations we recorded 16 cancellations of 65 planned TDMs (24.62%) in 10 of the 12 classes, of these three were due to the weather.

#### Planned and unplanned variation: pupil level adaptation

One of TDM Foundation’s core principles is to ‘keep it simple’ in order to ensure sustainability. However, teachers and other classroom staff typically adopted a range of incentives to keep children interested, including competitive activities (as above) and ‘collecting’ and recording miles in various ways. Although the scheme is designed to be inclusive, and in general, all children in a class did take part, gender differences in how the activity was undertaken were reported and observed. One was the greater tendency for girls to interact with each other while doing the mile:

the boys do tend to get into it a bit more than the girls. The girls often you’ll see them all linked arms walking around, having a little chat about it (Participant 11, Assistant Head Teacher)

Girls were more often observed moving during TDM holding hands and talking than the boys. However, our field notes also document them running, jogging and sprinting between these conversations.

## Discussion

Interventions such as TDM are difficult to evaluate in traditional public health terms with RCT or quasi-experimental designs. Pathways between ‘intervention’ and ‘outcome’ are long and not always linear [23]. This has a number of implications for evaluation. First, the health benefits that might accrue from an intervention may take many years to manifest. Second, effects of interventions may result from interactions between its components or those components and the context [31] making it difficult to attribute causality. Third, a key challenge for designing evaluations that are meaningful to potential evidence users is that primary outcomes may not be those that are most salient. Trial evaluations are typically powered on one primary outcome, whereas for many school staff a key benefit of this scheme, for example, was the way in which it integrated a number of perceived health and educational needs for their pupils.

However, given the local commitments to rolling out TDM as part of a broader strategy of obesity, and the focus of the TDM Foundation on particular health outcomes, it is perhaps not surprising that these health benefits were the anticipated outcomes mentioned most frequently. However, our findings also point to other benefits (e.g. cross school year peer relationships, teacher-student relationships, connection to other parts of the curriculum) that were important outcomes of the intervention. This assemblage of wellbeing benefits often facilitated variations in adoption and implementation. Few participants mentioned any potential negative effects, though the erosion of curricula time was a concern that was articulated in all schools. Where this was of particular concern, this, along with spatial constraints and, at times the weather, acted as key barriers that prevented TDM from taking place, and resulting in students not engaging in planned PA.

Of particular interest here in terms of outcomes is the focus, at least in this locality, on obesity. This is perhaps the outcome where there is less robust evidence for likely impact, given that TDM may increase PA without demonstrable effects on obesity, at least in the short to medium term, given the limited potential impact of an additional 15 min of PA for children. A systematic review that found ‘moderately’ strong evidence of the effect of obesity programmes in schools drew largely on US data, but with small effect sizes [32]. Recent high quality UK studies also reported similar small effect sizes but these were not statistically significant for outcomes including BMI, dietary and physical activity outcomes, as well as psychological measurements [33, 34]. Understanding this lack of apparent success is hampered by limitations in process evaluation literature, which tend to focus on what participants think about interventions [35]; and on a limited number of components, such as reach, dose and measures of fidelity [36]. Process evaluations on obesity have been less informative on what works (to encourage sustainability, as well as on outcomes) and why [37].

More generally, an intervention such as TDM can be thought of as an event within multiple interlinked complex systems [38] such as schools and local authorities: in this setting, this was explicitly recognised by the local authority who were taking a ‘whole system’ approach to obesity, which, as described earlier, involves a focus on PA, sugar intake and use of green space in the borough to promote healthy living. The limitations of RCT evidence for examining causality in such settings have been widely documented [23, 39], and are clear in this case. Disaggregating essential components to identify ‘what works’ may not be possible through cluster RCT methods, given the wide variety of methods of take up we have identified, and the complex interactions between school settings, the intervention and health states. This variation was evident at a number of levels. The local authority were supporting the scheme in their locality: others might not be, or might be using other materials or methods of support. At school level, many schools were implementing the scheme within broader programmes of healthy schools initiatives, including addressing food sales or lunch provision, as well as the adoption of other physical activity initiatives in some schools. There was also a sense from head teachers that schools had limited capacity to take part in multiple initiatives: if TDM was adopted, other potential schemes might not be adopted, which in turn may mean, for instance, that the focus is on PA, and less on food-related initiatives, such as Sugar Smart. In addition, there were concerns raised in each school about how the intervention would fit within existing curriculum and timetables that are ‘too full’, which has been identified elsewhere as a barrier [29]. Understanding if and how these decisions are made and what trade-offs are made at the level of the school is important for understanding intervention implementation.

There was considerable variation across classes in schools that had implemented the scheme. Even where teachers were taking out whole classes most days of the week, individual pupils were not necessarily getting an additional 15 min MVPA, with many not running, or not running unless encouraged in ‘competitive’ ways. The intervention, both in terms of what is being promoted and what is implemented, is also likely to change over time. TDM Foundation are amending their web site and informational material in part as a response to ongoing research findings, and are engaged in resourcing co-ordinators to assist schools with maintaining fidelity to core principles. There is also discussion around mandating the scheme in the light of evidence of its effectiveness for increasing PA in children. This would of course change the intervention: a scheme that schools are required to do might have different effects than one they choose, with a potentially higher likelihood of it displacing other activities.

There is increasing recognition of the ways in which context shapes the effects of interventions in complex systems [39, 40]. These effects are likely to have implications for health inequalities at a number of levels, each of which might (separately) mitigate or exacerbate existing inequalities in health outcomes. First, an intervention such as TDM might have differential effects on different population groups because of inequalities in uptake. We found no evidence of this within one borough, at least for the indicators available of deprivation levels and population mix of school populations (per cent eligible for free school meal, per cent in BME groups): schools that did agree to take part had no significant differences from those who did not. It is pertinent to note however that in a borough considered within the 20% most deprived in England [24], TDM uptake was slightly greater, though not statistically significant, across schools with a higher proportion of pupils having free school meals. Second, exposure by population group might differ if the intervention is implemented differently across the population. Although our rapid ethnography could not measure differences by gender, ethnicity or socio-economic status, we did identify some important ways in which these might shape the effects of the intervention in practice. For instance, at school level, schools with more constrained outdoor facilities (which in many settings include those in more disadvantaged areas) might be less likely to be able to sustain adherence; and at pupil level, we observed systematic gender differences in how much PA children were gaining from the intervention, whereby female students would be more likely to walk arm in arm with friends, compared to male students; and male children were more likely to define the outcomes of the intervention in terms of strength, and/or avoidance of weakness than females. Third, at the level of the individual, there is considerable evidence that the effects of specific activities are likely to differ depending on context, the subjective meaning attributed to the activity, and physiological status [41, 42]. Should these differences be systematically associated with social and demographic factors (such as the meaning of competitive activity for different genders, or the potential differential effects of exercise on children’s bodies who have not had access to breakfast [43]) the impact of the same exposure might be different. These factors are under-researched, but may account for the limited evidence for behavioural interventions on health equity [44]. Far more research is needed on the ways context interacts with interventions within complex systems, and the ways in which inequalities might emerge from those systems.

Recent guidance [16] suggests that descriptions of context are vital for reporting on public health interventions, to better understand how they work and why impacts may vary. We have described the context in one setting, a diverse London borough, and suggested that each school, and class, also has its own context. Methods for addressing the public health effects of interventions such as TDM, which will vary considerably by context, are underdeveloped. Experimental designs often do not illuminate how different causal conditions may operate in differing contexts to produce the intended outcomes: the successful implementation of an intervention. We suggest approaches that bring together quantitative and qualitative methods. This will help elucidate the configurations of conditions needed for implementation to be successful. To this end Qualitative Comparative Analysis (QCA) [45] might hold promise here as one way to explain causal complexity in context. QCA is a set-theoretic method, which examines casual complexity across a medium to large number of cases (between 10 and 60 +), whilst also being able to generalise across those cases [45, 46]. QCA analysis involves using Boolean algebra and formal logic to ask what conditions (alone or in combination with other conditions) across contexts are necessary or sufficient to produce some outcome [46]. QCA has been shown to be well placed to address concerns around complexity [47], though its use in the field of public health remains nascent [48].

### Strengths and limitations

This study was restricted to one borough in London and a relatively small number of schools. However, our sample of schools was relatively diverse, and sufficient to identify considerable variation in implementation. Our aim was to map this variation, and a rapid assessment was sufficient for this, but cannot provide an in-depth understanding of how and why interventions are adopted. More detailed ethnographic work would be needed to understand in detail what happened in schools when they took up the intervention. Our sample of schools and classes was also purposively rather than systematically selected, and drawn from those who volunteered. It is therefore less likely to represent schools uninterested in TDM, or in broader PA initiatives. We are therefore likely to have underreported challenges in implementation. The TIDieR-PHP checklist, although designed for use in describing interventions in evaluation studies, was a useful framework for structuring this study of implementation.

## Conclusions

The Daily Mile is, in principle, easy to adopt and has potentially positive impacts on children’s current and future health. However, evidence to date of its effects is likely to shed little light on what happens in practice in non-trial settings. This is important, as we have identified a variety of implementation practices in a non-trial setting, which have implications for future population health. This includes both variations in physical movements of the children taking part in each context, as well as variations in implementation, which result from, for example, various spatial (e.g. playground area) and temporal (e.g. time of year, time of the day and/or weather) constraints, as well as the presence or absence of other physical activities implemented within the school. All these adaptations are likely to shape the specific benefits and disbenefits for participants.

For complex interventions in public health, there have been calls for the development of more appropriate methods for both describing and evaluating interventions. This study found the TIDieR-PHP framework a useful checklist for describing TDM. In terms of future evaluation, TDM is typical of interventions for improving the health of the public, in that it is often implemented in the context of wider strategies, and in variable ways. Experimental and quasi-experimental methods have well-documented shortcomings for evaluating the causal effects of such complex interventions [23, 49]. Future evaluation of TDM, and similar interventions, could usefully draw on approaches such as QCA to explore both successful uptake and impact. Understanding this variability of intervention adoption and implementation is crucial to understand how it becomes adapted and whether or not we might define these adaptations as meeting the initial criteria of the intervention. Assessing interventions in this way acknowledges complexity in practice and has important implications for assessing intervention efficacy outcomes.

## Additional file

Additional file 1: Semi-structured interview and focus group schedules. (ZIP 69 kb)

## Data Availability

The datasets generated and/or analysed during the current study are not publicly available due to ongoing analysis (qualitative data) or confidentiality (Lewisham data). Anonymised interview transcripts will be deposited in due course, and will be available from the corresponding author on reasonable request from March 2020.

## Abbreviations

BME: Black or Minority Ethnic
BMI: Body Mass Index
LB: London Borough
MVPA: Moderate to vigorous physical activity
NCMP: National Child Measurement Programme
PA: Physical Activity
PE: Physical Education
QCA: Qualitative Comparative Analysis
TDM: The Daily Mile
