# Supplementary material for: The Daily Mile as a public health intervention: a rapid ethnographic assessment of uptake and implementation in South London, UK
Source: BMC Public Health. 2019 Aug 27;19:1167. doi: 10.1186/s12889-019-7511-9 (PMC6712825; doi:10.1186/s12889-019-7511-9)
Supplement: Supplementary file 1 — Semi-structured interview and focus group schedules. (ZIP 69 kb) [file 12889_2019_7511_MOESM1_ESM.zip › Focus Group Schedule_Parents and CarersR4.docx]

**Parents/Carers Focus Group**

Ask each participant to pick a pseudonym (write it on a name tag). Ask each person: # of child(ren) at the school, and year level.

Implementation in Classrooms:

1. Thinking back to when you first heard that The Daily Mile was going to be implemented in your school, could you please tell me:
   1. What did you think about it when you first heard of it?
   2. How did the school explain it to you?
2. Were you at all concerned about The Daily Mile when you first heard it was being implemented at the school?
   1. If so, what were your concerns?
3. What benefits do you think have come from your children participating in The Daily Mile at school?
4. Who do you think benefits most? Do you think there are different benefits for different children? (e.g. boys/girls, younger/older children, and/or if they come from different backgrounds?)
5. Is there anyone who you think does not benefit?

Parents TDM Group (where applicable):

1. Could you please tell me about The Daily Mile Parents group - what does it involve?
2. What is the best thing about participating?
3. What has been the hardest/most difficult thing about participating?
4. Tell me about yesterday’s Daily Mile
   1. Best/worst one so far this month?
5. Do you talk about your experiences of doing The Daily Mile with your children and your family at home? If so, what is discussed?
   1. Do you talk about it with anyone else outside your immediate family?

Overall Impression of TDM:

1. Overall do you think The Daily Mile has had an impact on your life? If so - in what ways?

Future:

1. Moving forwards do you think there is anything that could improve The Daily Mile for the future? If so, what might that be?
