# Supplementary material for: The Daily Mile as a public health intervention: a rapid ethnographic assessment of uptake and implementation in South London, UK
Source: BMC Public Health. 2019 Aug 27;19:1167. doi: 10.1186/s12889-019-7511-9 (PMC6712825; doi:10.1186/s12889-019-7511-9)
Supplement: Supplementary file 1 — Semi-structured interview and focus group schedules. (ZIP 69 kb) [file 12889_2019_7511_MOESM1_ESM.zip › Focus Group_Interview Schedule_ChildrenR4.docx]

Focus Group Schedule

**The Daily Mile**

Could you please explain in your own words what you think The Daily Mile is?

Why do you think your class is participating in The Daily Mile?

**Self-Reported Impact**

How does participating in TDM make you feel? (Do you enjoy it? Does it feel challenging/difficult? Why?)

Is it the same experience each time you do TDM? Or do you think it changes according to the day/time?

Do you think it helps you overall? If yes, why? If no, why not?

**Future Focus**

If you got to be in charge of running TDM for the day in your classroom what would you do? Is there anything you would change about it?
