# Supplementary material for: The Daily Mile as a public health intervention: a rapid ethnographic assessment of uptake and implementation in South London, UK
Source: BMC Public Health. 2019 Aug 27;19:1167. doi: 10.1186/s12889-019-7511-9 (PMC6712825; doi:10.1186/s12889-019-7511-9)
Supplement: Supplementary file 1 — Semi-structured interview and focus group schedules. (ZIP 69 kb) [file 12889_2019_7511_MOESM1_ESM.zip › Interview Schedule_Public Health StrategistsR4.docx]

Semi-Structured Interview Schedule (Public Health Strategists)

Intro

1. What is your role?
2. How did you come into contact with TDM project? How did you hear about it?
3. TDM – how do you see this fitting into the public health focus of Lewisham at this time?
   1. How does it fit into specific public health strategies 🡪 physical health/obesity strategy?
4. What do you see as your role in the implementation of TDM in the area? (e.g.: supporter/leader)

Logic Model – Anticipated benefits/challenges

1. Can you talk me through the logic model
   1. Do you think it is an accurate model of the way TDM will work?
   2. Do you think it misses anything?
   3. What do you think are the important pathways in this model?

Implementation

1. How do you think this will be received? (positive/negative)? Why?
2. What do you think will be some of the challenges to its implementation?
   1. Do you think this will differ between schools? Why?
3. What do you think are the costs associated with its implementation?
   1. Do you think this will differ between schools? Why?
4. What has been the reaction of schools to-date?

Future

1. What do you think it will look like in the future, in 5 – 10 years time?
   1. What would you like to see happen?
2. Thanks for your time – is there anything further you would like to add?
