# Supplementary material for: The Daily Mile as a public health intervention: a rapid ethnographic assessment of uptake and implementation in South London, UK
Source: BMC Public Health. 2019 Aug 27;19:1167. doi: 10.1186/s12889-019-7511-9 (PMC6712825; doi:10.1186/s12889-019-7511-9)
Supplement: Supplementary file 1 — Semi-structured interview and focus group schedules. (ZIP 69 kb) [file 12889_2019_7511_MOESM1_ESM.zip › Interview Schedule_Teachers R4.docx]

Semi-Structured Interview Schedule (Teachers)

Intro

1. What is your role?
2. How long have you been at the school?
3. How did you come into contact with the TDM project? How did you hear about it?
4. [Heads/A/Heads]
   1. Why did you decide to implement it in your school?
      1. Benefits?
      2. Concerns about implementing it?
5. How did people in your school react when it was first discussed as an intervention that would be implemented (teachers/students/parents).
   1. Was everyone receptive to it?
   2. Were you at all concerned about implementing it?

Implementation in Your School

1. Implementation:
   1. Tell me about yesterday’s Daily Mile
      1. Best/worst one so far this last fortnight?
   2. Have there been any challenges when implementing it? Any barriers? What was the hardest thing about implementing it?
   3. What didn’t you expect to work that did?
   4. What have been the benefits that you have seen so far?
   5. Do you think the benefits have been evenly distributed? Or do you think some children benefit more than others? (e.g. Gender/class). Please explain.
2. TDM Process:
   1. [Heads/A/Heads]
      1. Is there anything that you do to ensure it continues to take place in each classroom throughout the week? (I.e Do you have to promote it within the school?)
         1. If yes - How much time do you think you spend on this?
         2. How do you monitor it? Is there a record of how often the teachers in each classroom undertake it?
      2. Are there any other costs of running it? (E.g: ground maintenance)
         1. If so - how are these paid for? Do you have a budget allocated for this material?
   2. [All teachers]: How often is it implemented in your classroom? (Ideal vs. reality?)
   3. How do you ensure that when you undertake TDM that it does not clash with other teachers plans to run it at the same time? How much time does this take?
      1. Has there ever been a clash with other class schedules? What do you in these contexts? How do you negotiate this?
   4. From my understanding you do record The Daily Mile activity in each classroom. Could you explain this process to me? Who records it, and how? (Time taken)
   5. Parent TDM Group [where applicable]. Could you explain this.

Overall

1. Overall do you think it has benefited your classroom and students? Why?

TDM in the Future

1. What do you think The Daily Mile will look like in the future, in 3 - 5 years’ time?
   1. What would you like to see happen?
